# Effects of treatment with rituximab on microcirculation in patients with long-term systemic sclerosis

**DOI:** 10.1186/s13104-018-3994-1

**Published:** 2018-12-10

**Authors:** Verônica Silva Vilela, Bruno Rangel Antunes da Silva, Cláudia Henrique da Costa, Agnaldo José Lopes, Roger Abramino Levy, Rogério Rufino

**Affiliations:** State University of Rio de Janeiro, Rio de Janeiro, Brazil

**Keywords:** Systemic sclerosis, Microcirculation, Videocapillaroscopy, Rituximab

## Abstract

**Objective:**

To investigate the effect of rituximab on microcirculation in long-term SSc.

**Results:**

Four patients with diffuse SSc over 3 years of disease received rituximab cycles of two 1-g infusions every 6 months for 2 years. Videocapillaroscopy was performed at baseline, 12 months, and 24 months and semi-quantitative scoring of videocapillaroscopy abnormalities was performed and the microangiopathy evolution score (MES: range 0–9) was calculated. The mean disease duration was 5 years (range 3–15). On videocapillaroscopy, giant capillaries and hemorrhages remained stable from baseline to 24 months. Capillary loss, abnormally-shaped capillaries, and MES stabilized at 12 months and increased by 24.5% and 28% at 24 months. Rituximab improves microcirculation in long-term SSc. Stabilization and reduced progression of microcirculation abnormalities were achieved at 12 and 24 months, respectively.

**Electronic supplementary material:**

The online version of this article (10.1186/s13104-018-3994-1) contains supplementary material, which is available to authorized users.

## Introduction

Systemic sclerosis (SSc) is characterized by generalized vasculopathy and tissue fibrosis resulting from the interplay between the endothelium, fibroblasts, and inflammatory cells [1]. Microcirculation evaluated by nailfold capillaroscopy is correlated with the severity of organ involvement [2, 3]. Stem cell transplantation has been demonstrated to improve and rituximab to stabilize microcirculation in patients with SSc in the early stages of the disease [4, 5]. Rituximab is a monoclonal antibody that inhibits B lymphocyte proliferation and is effective in the treatment of several autoimmune diseases [6]. However, no study has evaluated the effects of rituximab on microcirculation in patients with long term SSc. Here, we report the effects of rituximab on microcirculation in four long-term SSc patients.

## Main text

### Methods

Observational study in patients with SSc using rituximab and analyzed the evolution of pulmonary function tests and videocapilaroscopy. Previously, the authors published a series of 10 patients with diffuse SSc (dSSc) with a disease duration > 3 years who received rituximab for the treatment of SSc skin and lung disease [6]. Among these, four patients underwent complete longitudinal pulmonary function tests and videocapillaroscopy with a semi-quantitative study performed at baseline, 12 months, and 24 months and were included in the present series. The study was approved by local ethics committee and all patients signed informed consent term. The six remaining patients continued to receive rituximab infusions but did not undergo sequential videocapillaroscopy exams and were not included in the present study. Pulmonary function tests were performed according to the American Thoracic Society and the results are expressed as percentages [7]. Chest computed tomographies were performed on a multislice equipment with high-resolution image acquisition.

Videocapillaroscopy exams were performed as follows: an optical probe with a 200-times magnification lens was connected to image software (Videocap 3.0; DS Medica). Four 1-mm images per digit were captured from digits 2–5 on both hands. Images were stored and coded to be evaluated blindly by an examiner who was unaware of the patient’s identification and clinical status. Semi-quantitative scoring of giant capillaries, hemorrhages, capillary loss, and abnormally shaped capillaries was performed according to previously described techniques [8]. Each abnormality was scored as follows: 0, absent; 1: present in ≤ 33%; 2: present in > 33 to ≤ 66%; 3: present in > 66% of the field. The microangiopathy evolution score (MES) was calculated from the sum of the scores of capillary loss, abnormally shaped capillaries and architectural derangement; values increase according to the severity of the abnormalities. Friedman’s test was used to calculate statistically significant differences between videocapillaroscopy abnormality values after 12 and 24 months. A p-value < 0.05 was considered statistically significant.

### Results

Three females and one male patient were included. Two patients were of African ethnicity and two were of European ethnicity; mean age was 43 years old (range 34–51). The mean disease duration was 5 years (range 3–15 years). Patients were refractory to previous cyclophosphamide or mycophenolate treatment. All were on stable dose of immunosuppressants; three received mycophenolate and one azathioprine. One patient also had autoimmune hepatitis; the remaining had no other diseases. All had moderate to severe restrictive lung syndrome and high-resolution tomography abnormalities (three ground glass opacities and one honeycombing).

**Fig. 1:**
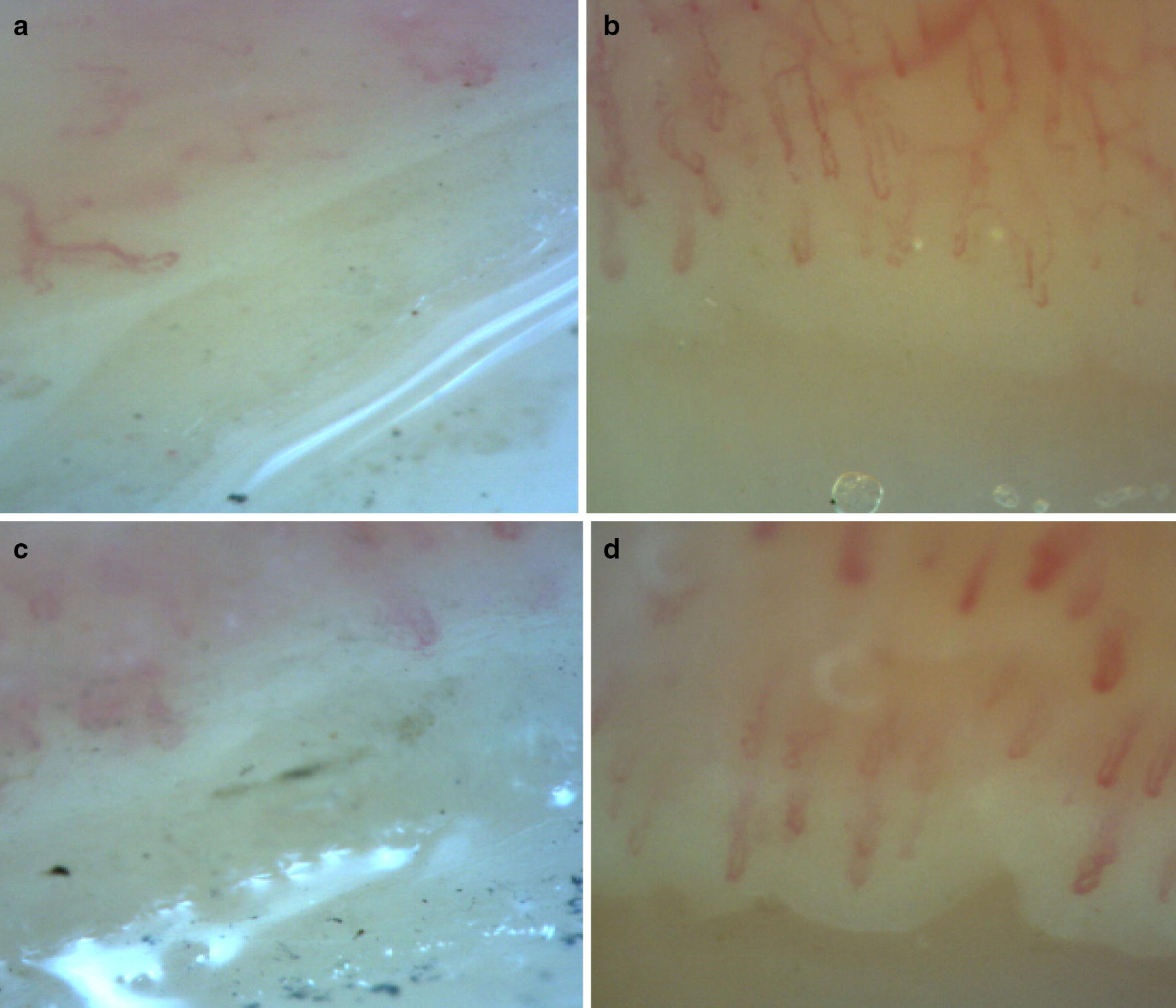
Findings in nailfold videocapillaroscopy. Nailfold videocapillaroscopy of patient 1 (**a**, **b**) and 2 (**c**, **d**) before and after 24 months treated-rituximab. **a**, **b** show abnormal organization and **b**, **d** return to normal organization of capillaries

**Fig. 2:**
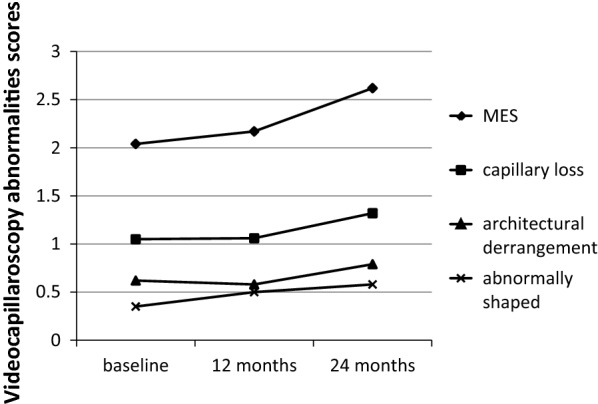
Capillary abnormalities evolution in SSc patients treated with rituximab

Table 1 and Fig. 1 show the videocapillaroscopy scores and pulmonary function at baseline, 12 months, and 24 months (Additional file 1). On videocapillaroscopy, giant capillaries and hemorrhage scores were low and remained stable from baseline to 24 months. Abnormally shaped capillaries increased at 12 months and stabilized at 24 months. Capillary loss was stable from baseline to time 1 and then increased by 24.5% (1.06–1.32) at time 2. Architectural derangement also stabilized at 12 months and then slightly increased at 24 months. The MES score increased from 2.04 at time 0 to 2.17 at time 1 and 2.62 at time 2 (28% increase). Figure 2 shows the evolution of capillary abnormalities at the three time points. No changes associated with capillary abnormalities across time achieved statistical significance.

**Table 1:** Pulmonary function test and videocapillaroscopy abnormalities mean values at baseline, 12 months and 24 months

Variable	Time 0	Time 1	Time 2	p*
FVC (mean %)	65.0	64.0	68.5	0.65
DLco (mean %)	52.3	53.5	60.0	0.06
Giant capillaries (score 0–3)	0.03	0.02	0.01	0.66
Hemorrhages (score 0–3)	0.10	0.06	0.06	> 0.99
Capillary loss (score 0–3)	1.06	1.05	1.32	0.18
Abnormally shaped capillaries (score 0–3)	0.35	0.50	0.58	> 0.99
Architecture (score 0–3)	0.62	0.59	0.79	0.66
MES	2.04	2.17	2.62	0.93

Observation: The non-random sample was small to demonstrate statistical differences. *It was used the Friedman test which is non-parametric statistical test

*FVC* forced vital capacity, *DL*_*CO*_ diffusion capacity of the lung for carbon monoxide, *MES* microangiopathy evolution score, range 0 (absence of the abnormality) to 3

With respect to pulmonary function, forced vital capacity stabilized at time 1 and slightly improved at time 2. Measurement of diffusion capacity for carbon monoxide could not be performed in one patient. Therefore, the mean value at 12 months was not considered. However, values increased from a mean of 53% at time 1 to 60% at time 2 (Table 1).

### Discussion

This study investigated the beneficial effects of rituximab on microcirculation in patients with long-term SSc who received intensive regular rituximab infusions mainly for the treatment of moderate to severe interstitial lung disease. We also assessed lung function stabilization.

Previous studies have evaluated the effects of disease evolution and various treatments on microcirculation in SSc using semi-quantitative scoring systems for videocapillaroscopy. In patients with long-term disease, Sulli et al. reported that a significant worsening of microcirculation abnormalities occurred with disease progression [8]. Miniati et al. reported that in patients with a short disease duration, stem cell transplantation significantly improved and cyclophosphamide stabilized microcirculation at 24 months [4]. Specifically, MES had an impressive 65% improvement (decreasing from 2.57 to 1.65) in patients treated with stem cell transplantation, whereas patients who received cyclophosphamide had stable scores. Only two patients had restrictive pulmonary syndrome and no patient had lung fibrosis. More recently, Smith et al. reported capillary loss score stabilization at 2 years after two infusions of 1-g cyclophosphamide at baseline and 6 months in patients with early dSSc.

In this study, patients had a profile similar to those reported in the study of Sulli et al. [8], with long-term disease, more organ damage, and similar baseline capillary abnormalities. With rituximab infusions, the patients experienced microcirculation stabilization at 12 months. At this time, the capillary loss score was unchanged (1.05 at baseline and 1.06 at 12 months) and an MES score increase of just 6.3% occurred. This was similar to that achieved in the study of Smith et al. [5] in patients with early SSc. At 24 months, the patients had 24.5% and 28.3% mean increases in capillary loss score and MES, respectively. This was better than the 24-month values of capillary loss and MES of 25% and 40%, respectively, reported by Sulli et al. [8].

In this study, we believe that microcirculation stabilization at 12 months and a reduction in the rate of progression at 24 months was related to treatment with regular rituximab infusions. Thus, even in patients with long-term disease, treatment with rituximab may decrease the progression of microcirculation abnormalities. Simultaneous improvement in lung function occurred, which was similar to the results of Lepri et al. [9] and Moazedi-Fuerst et al. [10], who reported pulmonary function improvements in SSc patients receiving regular rituximab infusions. In conclusion, SSc is a serious disease with limited therapeutic options.

Rituximab appears to improve microcirculation in long-term SSc.

## Limitations

This study has a very small number of patients, which hinders the statistical evaluation and is from a single research center. The patients were inserted by loss of lung function, i.e. with more severe patients.

## Additional file

**Additional file 1.** videocapillaroscopy data. videocapillaroscopy semiquantitative (0-3) score. This file discloses data on the mean values of the videocapillaroscopy scores of capillary loss, abnormally shaped capillaries, architectural derangement and MES score on individual patients at different time points and their mean values.

## Abbreviations

SSc: systemic sclerosis
dSSc: diffuse systemic sclerosis
MES: microangiopathy evolution score
